# Characteristics of epigenetic aging across gestational and perinatal tissues

**DOI:** 10.1186/s13148-021-01080-y

**Published:** 2021-04-29

**Authors:** Linda Dieckmann, Marius Lahti-Pulkkinen, Tuomas Kvist, Jari Lahti, Peter E. DeWitt, Cristiana Cruceanu, Hannele Laivuori, Sara Sammallahti, Pia M. Villa, Sanna Suomalainen-König, Johan G. Eriksson, Eero Kajantie, Katri Raikkönen, Elisabeth B. Binder, Darina Czamara

**Affiliations:** 1 Department of Translational Psychiatry, Max Planck Institute of Psychiatry, München, Germany; 2 International Max Planck Research School for Translational Psychiatry, München, Germany; 3 Department of Psychology and Logopedics, Faculty of Medicine, University of Helsinki, Helsinki, Finland; 4 National Institute for Health and Welfare, Helsinki, Finland; 5 Centre for Cardiovascular Science, Queen’s Medical Research Institute, University of Edinburgh, Edinburgh, UK; 6 Section of Informatics and Data Science, Department of Pediatrics, University of Colorado School of Medicine, Aurora, CO USA; 7 Institute for Molecular Medicine Finland, HiLIFE, University of Helsinki, Human Genetics, Helsinki, Finland; 8 Medical and Clinical Genetics, University of Helsinki and Helsinki University Hospital, Helsinki, Finland; 9 Department of Obstetrics and Gynecology- Faculty of Medicine and Health Technology, Tampere University Hospital and Tampere University, Tampere, Finland; 10 Children’s Hospital, Helsinki University Hospital and University of Helsinki, Helsinki, Finland; 11 Department of Child and Adolescent Psychiatry, Erasmus MC, Sophia Children’s Hospital, Rotterdam, The Netherlands; 12 Department of Obstetrics and Gynecology, Helsinki University Central Hospital, Helsinki, Finland; 13 Hyvinkää Hospital, Helsinki and Uusimaa Hospital District, Hyvinkää, Finland; 14 Folkhälsan Research Center, Helsinki, Finland; 15 Department of Obstetrics & Gynaecology and Human Potential Translational Research Programme, Yong Loo Lin School of Medicine, National University of Singapore, Singapore, Singapore; 16 Singapore Institute for Clinical Sciences, Agency for Science, Technology and Research (A*STAR), Singapore, Singapore; 17 Faculty of Medicine, PEDEGO Research Unit, MRC Oulu, Oulu University Hospital and University of Oulu, Oulu, Finland; 18 Department of Clinical and Molecular Medicine, Norwegian University of Science and Technology, Trondheim, Norway; 19 Department of Psychiatry and Behavioral Sciences, School of Medicine, Emory University, Atlanta, GA USA

**Keywords:** Epigenetic clocks, Early development, Epigenetic age, Perinatal tissues, Cord blood, Placenta, Chorionic villi

## Abstract

**Background:**

Epigenetic clocks have been used to indicate differences in biological states between individuals of same chronological age. However, so far, only few studies have examined epigenetic aging in newborns—especially regarding different gestational or perinatal tissues. In this study, we investigated which birth- and pregnancy-related variables are most important in predicting gestational epigenetic age acceleration or deceleration (i.e., the deviation between gestational epigenetic age estimated from the DNA methylome and chronological gestational age) in chorionic villus, placenta and cord blood tissues from two independent study cohorts (ITU, n = 639 and PREDO, n = 966). We further characterized the correspondence of epigenetic age deviations between these tissues.

**Results:**

Among the most predictive factors of epigenetic age deviations in single tissues were child sex, birth length, maternal smoking during pregnancy, maternal mental disorders until childbirth, delivery mode and parity. However, the specific factors related to epigenetic age deviation and the direction of association differed across tissues. In individuals with samples available from more than one tissue, relative epigenetic age deviations were not correlated across tissues.

**Conclusion:**

Gestational epigenetic age acceleration or deceleration was not related to more favorable or unfavorable factors in one direction in the investigated tissues, and the relative epigenetic age differed between tissues of the same person. This indicates that epigenetic age deviations associate with distinct, tissue specific, factors during the gestational and perinatal period. Our findings suggest that the epigenetic age of the newborn should be seen as a characteristic of a specific tissue, and less as a general characteristic of the child itself.

**Supplementary Information:**

The online version contains supplementary material available at 10.1186/s13148-021-01080-y.

## Background

DNA methylation (DNAm) is considered a biomarker of aging, with the potential to uncover differences in the biological age between individuals of the same chronological age [1, 2]. Epigenetic clocks make use of individual methylation patterns to estimate epigenetic age, and deviations between chronological and epigenetic age can be used to calculate relative epigenetic age acceleration (epigenetic age older than chronological age) and epigenetic age deceleration (epigenetic age younger than chronological age) in underlying tissues [3–5]. Commonly, these measures of epigenetic aging are calculated as the residuals of regressing predicted epigenetic age on chronological age, also called epigenetic age acceleration residuals (EAAR).

Epigenetic age acceleration has been linked to differences in long-term health outcomes and all-cause mortality in adults [6–8]. Changes in DNA methylation status have been proposed to be a mechanism by which environmental influences may become biologically embedded [9–11], and in fact, epigenetic age has been shown to be moderated by environmental exposures and lifestyle risk factors, such as education, body mass index (BMI), nutrition and smoking, among others [12–14]. These findings underscore the utility of epigenetic clocks as a means to investigate aging processes in general, and how these relate to environmental exposures and negative health outcomes or diseases. However, despite the sensitivity to and importance of epigenetic programming during the early developmental period [15, 16], studies investigating epigenetic age during the earliest phase of life are still underrepresented.

Various epigenetic clocks have been developed, for different tissues, ages and purposes [7, 17–19]. Specifically for the gestational period, two clocks for cord blood [20, 21], as well as two clocks for placental tissue [22, 23] have been established. For gestational epigenetic age estimation in cord blood, both the Knight [20] and Bohlin [21] clocks have been used in previous studies. Applying Knight’s clock, epigenetic age deceleration has been linked to exposure to negative pregnancy environments including insulin-treated gestational diabetes mellitus in a previous pregnancy, maternal history of depression and greater antenatal depressive symptoms, maternal Sjögren’s syndrome and a prenatal adverse environment assessed with the cerebroplacental ratio, as well as negative prospective child outcomes such as early childhood psychiatric problems [24–26]. These findings, together with the observation that epigenetic age acceleration was related to a lower need of respiratory interventions, led to the hypothesis that gestational epigenetic age deceleration may be related to a lower developmental maturity [27]. This seems to be supported by results from the Bohlin clock, where epigenetic age acceleration has been associated with higher birth weight and length [28], as well as higher head circumference, vaginal delivery, male sex and higher maternal pre-pregnancy BMI [29]. However, epigenetic age acceleration has also been associated with lower birth length, a lower 1-min Apgar score, fetal demise in a previous pregnancy, maternal preeclampsia, maternal age over 40 years at delivery and treatment with antenatal betamethasone [24], thus not supporting this hypothesis. Despite that, it should be noted that it was recently shown that CpGs relevant for epigenetic aging in general were linked to developmental processes [30].

Regarding placental tissue, Mayne et al. [23] found epigenetic age acceleration to be associated with early onset preeclampsia. Another study using Mayne’s clock reported a link between higher epigenetic age acceleration in the placenta and lower fetal weight and other growth measures among males, but increased fetal weight and growth among females [31]. Furthermore, placental epigenetic age deceleration has been associated with maternal weight gain during pregnancy, and for mothers of male offspring with pre-pregnancy obesity and higher blood pressure [32]. So far, to our knowledge, no comparable studies were performed with the placental clock presented by Lee [22]. Although research in this field is growing since the development of perinatal tissue clocks, studies considering different available clocks, and various birth- and pregnancy-related variables in a combined fashion, are largely lacking. More studies are needed to achieve a better understanding of the associations of epigenetic age deviations in perinatal tissues with exposures and outcomes, and especially how these deviations compare across tissues. Such insights are critical to gain a better knowledge of aging and developmental processes during the earliest phase in life and may help to find intervention strategies in the long term.

The aim of this explorative study was to I) identify factors among various birth- and pregnancy-related variables which are most predictive of epigenetic (DNAm) age acceleration or deceleration in first trimester placental tissue derived from chorionic villus sampling (CVS), term placenta and cord blood collected at birth, and II) characterize the relationship between epigenetic age deviations across gestational and perinatal tissues from the same individuals.

We used data from two independent Finnish cohorts, the intrauterine sampling in early pregnancy study (ITU), and the prediction and prevention of preeclampsia and intrauterine growth restriction study (PREDO). We assessed gestational epigenetic age in early-pregnancy CVS samples, and cord blood and fetal-side or decidual-side placental tissue sampled at birth (ITU: 693 individuals and 1176 tissue samples from CVS and/or term fetal placenta and/or cord blood, PREDO: 966 individuals and 1083 samples from term decidual placenta and/or cord blood). We calculated the epigenetic age with both available clocks per tissue, and applied Bohlin’s clock for cord blood [21] and Lee’s clock for placenta [22], based on better accuracy metrics of these clocks in the data sets. The predictive power of several birth- and pregnancy-related variables for a higher or lower deviance between estimated epigenetic and chronological gestational age (GA) was tested in every tissue separately, and finally, cross-tissue correlations were evaluated.

To the best of our knowledge, this is the first study of epigenetic age in CVS samples, and across multiple gestational/perinatal tissues assessed from the same individuals.

## Methods

### Study populations

The intrauterine sampling in early pregnancy study (ITU) consists of Finnish women and their children born between 2012 and 2017. The women were recruited through the national voluntary prenatal screening program for trisomy 21, available for all pregnant women in Finland free of charge.

ITU study comprises two study arms. 1) Women in the chromosomal testing arm had been referred to the Helsinki and Uusimaa Hospital District Fetomaternal Medical Center (FMC) because they had an increased risk of fetal chromosomal abnormalities based on routine serum and ultrasound screening, age, and patient history. They underwent fetal chromosomal testing (CVS, amniocentesis, or noninvasive prenatal testing) at FMC. Women were informed about the study during FMC visits. If the chromosomal test indicated no fetal chromosomal abnormalities, those who had expressed interest in participating were contacted for final recruitment. Those whose chromosomal test results suggested a fetal chromosomal abnormality were not recruited. 2) Women in the no chromosomal testing arm underwent the same routine screening for fetal chromosomal abnormalities. Based on their serum and ultrasound screening, age and patient history, they were *not* referred to FMC for fetal chromosomal testing. The women were informed about ITU when attending the routine screening at maternity clinics. Women who expressed interest in participating were contacted for final recruitment into this study. Both study arms provided placenta and cord blood samples for this study. CVS tissue was only acquired from the chromosomal testing arm participants who underwent CVS sampling at FMC.

The Prediction and Prevention of Preeclampsia and Intrauterine Growth Restriction (PREDO) study is a longitudinal multicenter pregnancy cohort study of Finnish women and their singleton, born-alive children between 2006 and 2010 [33]. The recruitment took place when the mothers attended their first ultrasound screening in early pregnancy. The PREDO comprises two subsamples: the clinical arm recruited based on having risk factors for preeclampsia and intrauterine growth restriction, and the epidemiological arm recruited from study hospitals independently of the presence of risk factors.

All participating women in both cohorts signed written informed consent forms for them and their children to participate in the study. The consents enabled linkage of nationwide health register data using unique personal identification numbers assigned to all Finnish citizens and permanent residents since 1971.

### Sampling of biological tissues

In ITU, CVS samples were taken based on medical indication between 10–15 weeks of gestation. Any CVS surplus tissue, not needed for clinical purposes, was immediately stored at − 80℃.

Placenta samples were collected after birth and midwives/trained staff took nine-site biopsies (within maximum 120 min after delivery for ITU, and maximum 90 min after delivery for PREDO). In ITU, placental samples were taken from the fetal side of the placenta, at 2–3 cm from umbilical cord insertion and the biopsies were first stored at + 5 °C and then at − 80 °C. In PREDO, samples were taken from the decidual side of the placenta and immediately stored at − 80 °C.

For both ITU and PREDO, cord blood samples were taken immediately after birth by a midwife.

### DNA methylation

From the collected samples, DNA was extracted according to standard procedures. Methylation analyses were performed at the Max Planck Institute of Psychiatry in Munich, Germany. We aimed to use 400 ng DNA for bisulfite-conversion with the EZ-96 DNA Methylation kit (Zymo Research, Irvine, CA). For n = 71 CVS samples, this was not feasible and we used lower amounts of DNA (from 48 ng upward). We saw no relation between the amount of DNA and our quality control measures. DNA samples were run on the Illumina Infinium MethylationEPIC array (Illumina, San Diego, USA), and for an additional set of cord blood samples from PREDO on the Infinium HumanMethylation450 BeadChip (Illumina, San Diego, USA). In total, methylation levels were assessed in n = 277 CVS samples, n = 500 placental samples and n = 437 cord blood samples from ITU (all assessed on the EPIC array), and in n = 140 placental samples and n = 160 cord blood samples (EPIC array) and an additional n = 876 cord blood samples processed with the 450 K array from PREDO.

Preprocessing of all methylation samples was conducted using the same pipeline [34] and the R package *minfi* [35]. Scan intensity signals as stored in.idat files were loaded into R and transformed into beta-values.

Samples with a mean detection p value > 0.05 were excluded (ITU: eight for CVS, one for placenta, none for cord blood; PREDO: none for placenta, three for cord blood run on EPIC, three for cord blood run on 450 K). Additionally, we excluded samples presenting with distribution artifacts in raw beta-values (ITU: five for CVS, nine for placenta, one for cord blood; PREDO: none for placenta, three for cord blood run on EPIC, eight for cord blood run on 450 K), as well as samples showing sex mismatches between estimated sex (using the *getSex* function) from methylation data and confirmed phenotypic sex (ITU: none for CVS, four for placenta, one for cord blood; PREDO: one for placenta, four for cord blood run on EPIC, n = 19 for cord blood run on 450 K). Further n = 20 samples needed to be excluded from the PREDO cord blood data set run on the 450 K array due to technical artifacts. Beta-values were normalized using stratified quantile normalization [36], followed by BMIQ [37]. Afterward, beta-values were transformed into M values, and batch-effects were removed using Combat [38]. For this, we computed a principal component analysis (PCA) on the M values and checked which batches were most strongly associated with the principal components. The strongest batches for the respective data set were iteratively removed (for ITU these were plate, array and slide in CVS; plate, slide and array in placenta; and plate and array in cord blood; for PREDO these were plate, array and slide in placenta; plate and array in cord blood run on the EPIC array; plate and array in cord blood run on the 450 K array). Corrected M values were re-transformed into beta-values.

In a next step, we applied MixupMapper [39] to the genotype and methylation data to check for possible sample mix-ups. Mix-ups occurred solely in the PREDO cord blood data set from 450 K array and n = 12 samples were removed.

For cord blood samples, contamination with maternal blood was tested [40] and samples identified as contaminated were excluded from further analyses (ITU: nine for cord blood; PREDO: one for cord blood run on EPIC, n = 19 for cord blood run on 450 K).

The final data sets from ITU comprise 264 samples from CVS, 486 samples from placenta and 426 samples from cord blood. The final data sets from PREDO comprise 139 samples from placenta, 149 samples from cord blood from EPIC and 795 samples from cord blood from 450 K.

**Fig. 1:**
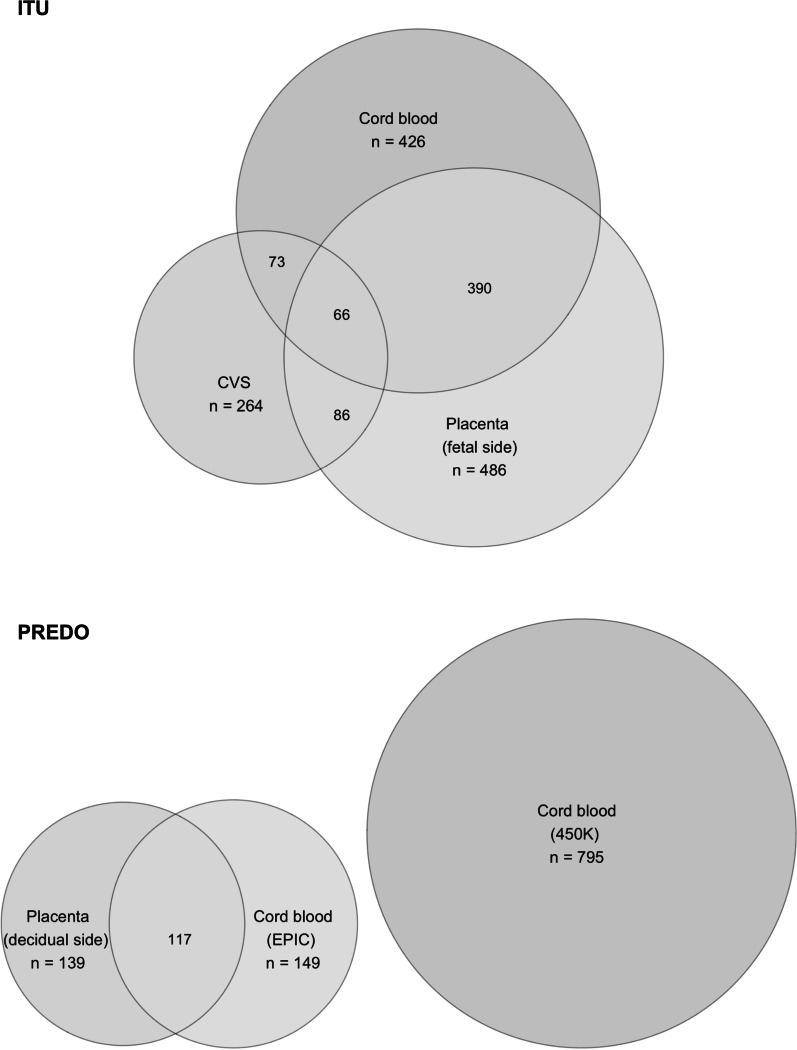
Sample overview for both cohorts used. Samples with methylation data available from different tissues in ITU and PREDO. In total, the ITU data set comprised 693 individuals after QC, with 264 CVS, 486 fetal placenta and 426 cord blood samples. For some individuals, samples were available from several tissues, indicated by overlapping circles. The final PREDO data set comprised 171 individuals after QC processed with the EPIC array, and additional 795 individuals processed with the 450 K array. From the EPIC data, 139 samples were available from placenta, and 149 samples from cord blood. The number of individuals with data from both tissues is again illustrated by the overlapping circles

The final data sets with sample sizes are illustrated in Fig. 1.

### Gestational epigenetic and chronological age

Gestational epigenetic age (DNAm GA) was estimated for cord blood using both the methods proposed by Knight et al. [20] and Bohlin et al. [21]. For Knight’s clock, the estimation of DNAm GA was based on the methylation profile of 142 from the original 148 CpGs, due to the lack of 6 CpGs on the EPIC array. Excluding the missing CpGs from the calculation was also recommended by the authors [20], who reported a high correlation between estimates from the full and reduced epigenetic age predictor. For the calculation of DNAm GA with Knight’s clock, we applied the script provided by the authors on the raw, un-normalized data. For Bohlin’s clock, the estimation of DNAm GA was constituted on 88 from 96 CpGs, also following from differences between the underlying arrays. DNAm GA in chorionic villi and placenta samples was estimated using 558 CpGs proposed by Lee et al. [22]. Additionally, we estimated DNAm GA using 57 CpGs available on the EPIC array from the original 62 CpGs determined by Mayne et al. [23]. A list of the CpGs missing on the EPIC array for the respective clocks can be obtained from Additional file 1.

Child chronological gestational age (GA) was based on fetal ultrasound, performed before 24 + 0 weeks of gestation and extracted from the Finnish Medical Birth Register (MBR).

### Cell-type composition estimations

Cell-type composition into seven cell types (nucleated red blood cells, granulocytes, monocytes, natural killer cells, B cells, CD4( +) T cells and CD8( +) T cells) in cord blood was estimated in *minfi* based on the approach proposed in Gervin, Salas [41].

Cell-type composition into six cell types (nucleated red blood cells, trophoblasts, syncytiotrophoblasts, stromal, Hofbauer, endothelial) in CVS and placenta was estimated using a recently published reference [42] and implementation within the R package *planet*, by applying the robust partial correlation algorithm [43].

The mean estimated cell types for every data set are given in Additional file 2.

### Genotyping and ancestry-related information

Genotyping was performed on Illumina GSA-24v2-0_A1 arrays for ITU, and on Illumina Human Omni Express Arrays for PREDO, according to the manufacturer’s guidelines (Illumina Inc., San Diego, CA). Quality control was performed in Plink 1.9 [44] and R [45]. DNA was extracted from cord blood, if available, otherwise placental tissue was used. SNPs with a minor allele frequency below 1%, a call rate below 98%, or with deviation from Hardy–Weinberg-Equilibrium with a p value < 1 × 10^–05^ were removed from the analysis. Furthermore, SNPs mapping to multiple locations as well as duplicated variants were removed. Individuals with a genotype call-rate below 98% were also excluded. Any pair of samples with IBD estimates > 0.125 was checked for relatedness. Within PREDO, high IBD estimates could be resolved due to shared ethnical origin of these individuals except for one pair. From this pair, we excluded one sample from further analysis. In ITU, seven samples were removed. Furthermore, individuals showing discrepancies between phenotypic and genotypic sex (one in PREDO, none in ITU) were removed.

To retrieve ancestry-related information, we performed multi-dimensional scaling (MDS) analysis on the IBS matrix of quality-controlled genotypes [46], where available. Outliers, defined as samples presenting with a position on any of the first ten axes of variation deviating more than four standard deviations from the respective axis’ mean, were iteratively removed until no more outliers were detected. Afterward, individuals presenting with heterozygosity values more than four standard deviations away from the mean heterozygosity were also iteratively removed (none in PREDO, two in ITU). The first two components were extracted and included as covariates in following analyses. In total, ancestry-related information for ITU was available from 587 of the 693 individuals included in our analyses, for 148 of the 171 individuals from PREDO with methylation data from the EPIC array, and for 787 of the 795 individuals from PREDO with methylation data from the 450 K array.

### Birth- and pregnancy-related variables

We included 14 birth- and pregnancy-related variables which were available for all tissues in both data sets.

In both cohorts, child sex, birth weight (kg), birth length (cm) and birth head circumference (cm) were measured at birth and data were extracted from the MBR. Maternal age (years) at delivery, early pregnancy BMI, calculated from weight and height verified by measurement at the first antenatal clinic visit, smoking during pregnancy (yes or no), parity (primiparous or multiparous), mode of delivery (unaided vaginal delivery or aided delivery, including breech, forceps, vacuum, cesarean section), and induction of labor (yes or no) were obtained from the MBR. Diagnoses of maternal diabetes disorders (yes for both types I & II, as well as gestational diabetes [ICD-10: E08-E14, O24] or none) until childbirth, and hypertensive pregnancy disorders such as gestational hypertension or pre-eclampsia in the current pregnancy (yes [ICD-10: O10-O14] or no), were extracted combining data from the MBR and the Finnish nationwide Care Register for Healthcare (CRHC). The CRHC carries primary and subsidiary diagnoses of all inpatient and outpatient hospital visits in Finland and from all treatments in specialized public outpatient care in Finland. In PREDO, the CRHC and MBR diagnoses were confirmed by a clinical jury, which comprised two physicians and a study nurse. Diagnoses of any maternal mental or behavioral disorder [ICD-8 and ICD-9: 290–319; ICD-10: F00-F99] until child birth were extracted from the CRHC. Alcohol use during early pregnancy was reported by the mothers (for PREDO around gestational week 12–13, for ITU around gestational week 20).

### Statistical analyses

All statistical analyses were conducted in R version 4.0.2 [45].

#### Measuring deviations between epigenetic age and chronological age

In previous studies, two measures of epigenetic age acceleration were considered, one based on the raw difference between DNAm age and chronological age, and the other calculated as the residuals from regressing DNAm age on chronological age. While the former provides a more intuitive interpretation and the investigation of the disjunct effects of epigenetic age, the latter is preferable in terms of its statistical properties—it addresses the dependency of age acceleration on chronological age and is comparable across studies. Therefore, we defined the deviation between gestational epigenetic age (DNAm GA) and chronological gestational age (GA) in all statistical models as the residuals (*epigenetic age acceleration residuals, EAAR*) resulting from regressing DNAm GA on GA, cell types of the respective tissue and the first two ancestry-related components derived from genotypic information. A positive EAAR value suggests faster biological aging, i.e., a higher epigenetic than chronological age (epigenetic age acceleration), and a negative EAAR value suggests slower biological aging, i.e., a lower epigenetic than chronological age (epigenetic age deceleration).

#### Identification of factors impacting epigenetic age acceleration/deceleration per tissue

**Fig. 2:**
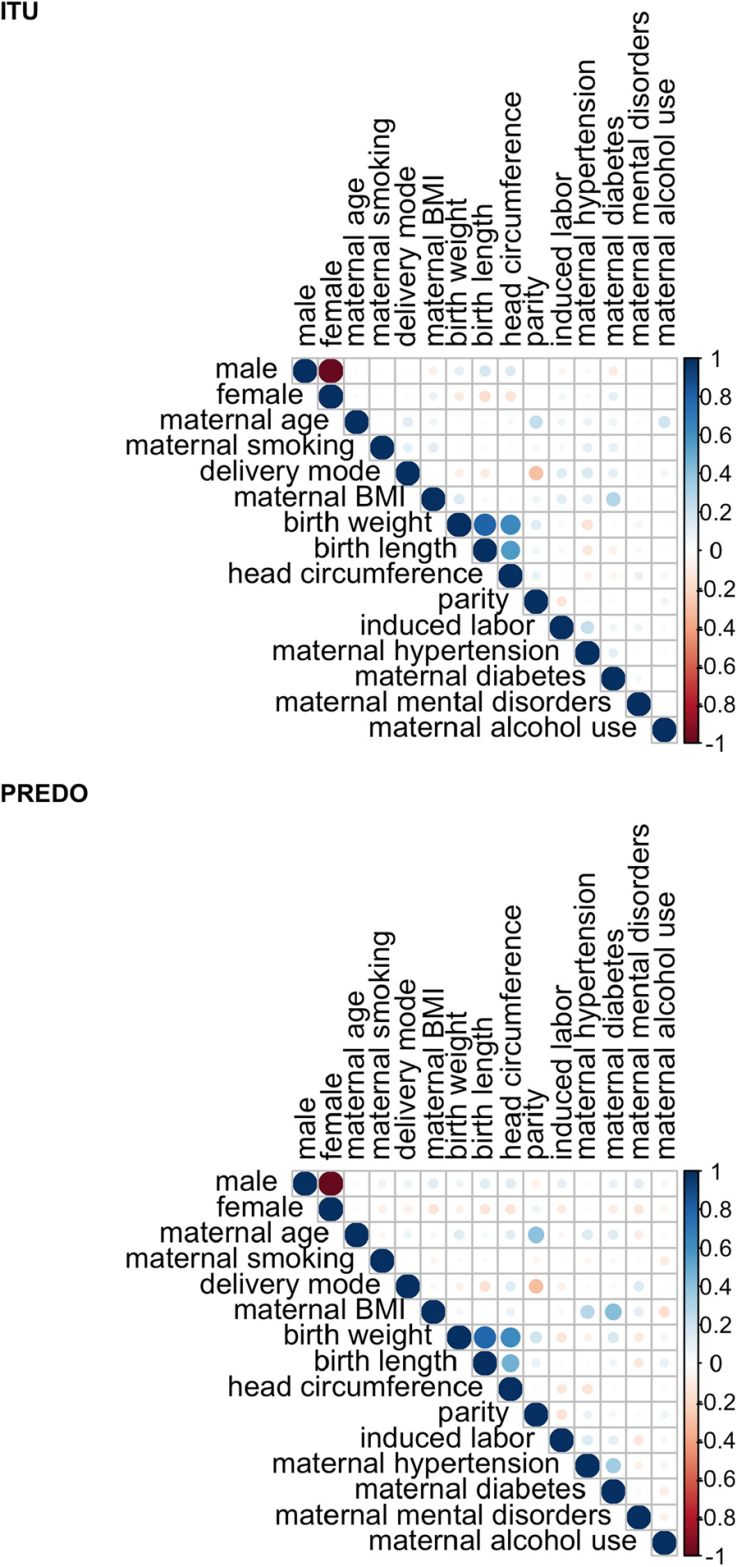
Pearson correlations among the predictor variables for ITU (N = 693) and PREDO (N = 171)

Our aim was to identify those of the available birth- and pregnancy-related variables that were most predictive of higher or lower EAAR. Without sufficient prior information enabling a hypothesis-driven selection of predictors, we decided to choose an appropriate data-driven variable selection method. Further, to reduce confounding effects, all predictors were evaluated in one model and correlations between predictors (see Fig. 2 for an overview) were considered, using elastic net regressions combined with a bootstrap approach for an evaluation of robustness. Separate models were run for all tissues, and cohorts, including placental models from ITU (fetal) and PREDO (decidual).

For every model, all predictor variables and the outcome variable (EAAR) were z-standardized, to ensure that the penalization was fair to all regressors and to enable the interpretation of the size of coefficients in terms of importance. Further, only complete observations were included. Bootstrapping was performed with 1000 bootstrap samples on every input data set. On every bootstrap sample, an elastic net regression was fitted with the R package *ensr* [47], which is built on *glment* [48]. Hyperparameters were selected by tenfold cross-validation, default lambda values (n = 100) and a sequence of 11 alpha values between 0 and 1 (by steps of 0.1). The output was grouped by bootstrap and number of non-zero coefficients (nzero) resulting from the different alpha levels. Of these, the models with minimum mean cross-validation error (cvm) with the respective parameters were chosen as best models (for every bootstrap and number of nzero). Afterwards, the percentage of a variable being not zero was calculated over all bootstrap samples for every number of nzero. Further, the median cvm over the bootstrap samples was plotted for every number of nzero. At this point, a final number of nzero must be chosen, with a necessary trade-off between model complexity and error (bias-variance tradeoff [49]). To aid the decision of non-zero coefficients in smoothly decreasing curves, we looked at the elbow in the plot of the median cvm for every nzero, by using a function drawing a straight line from the first to the last point of the curve and finding the data point farthest away from this line. This point can indicate the position of most decreasing cvm. The respective number of nzero can be used for further analysis steps. Due to the bootstrapping procedure, there was still variation in the variables and their coefficients in the final model. If a predictor was selected in > 75% of the bootstrap samples, we declared it as sufficiently stable and important. This approach for variable selection was referred to as *variable inclusion probability (VIP)* in a previous paper, where the authors used a comparable method for neuroimaging data [50]. The median coefficients and 95% confidence intervals over bootstraps, when the variable was not zero, were also calculated. An illustration of the analysis steps is given in Additional file 3.

#### Replication of cord blood findings between cohorts

To evaluate the predictability of the chosen predictors in ITU in the PREDO data set, the median coefficients of the identified variables in ITU were used to predict EAAR in PREDO. The one-tailed Pearson correlation between predicted and observed EAAR values was calculated. Additionally, we performed the same elastic net analysis applied in ITU cord blood data independently in the PREDO cord blood data sets to confirm that the directions of associations are consistent with those observed in ITU (Additional file 4).

#### Cross-tissue analyses

Pearson correlations for both DNAm GA and EAAR were calculated between cord blood and placenta, as well as between CVS and placenta and CVS and cord blood, for persons with multiple tissue sample available. To test if there are significant differences in mean age acceleration or deceleration between the tissues, we applied paired Student’s t tests, or paired Wilcoxon signed-rank test, between EAAR values of the respective tissues.

#### Complementary analyses

It has been reported that child sex can be an important factor when considering how placenta function is affected by direct environmental factors [51], and sex differences in epigenetic aging have been reported [31, 32]. Therefore, we repeated our analyses in placenta stratified by sex as described in Additional file 5.

Additionally, information about maternal alcohol use during pregnancy was only available in 580 samples from ITU, 153 samples from the EPIC array in PREDO and 693 samples from the 450 K array in PREDO.

To avoid larger reductions in sample size for the remaining predictors, we did not include this variable in the main models per tissue, but provide it in supplementary analyses (Additional file 6).

## Results

A summary of characteristics of the available data sets is given in Table 1.

**Table 1:** Characteristics of available data sets: *Mean (SD*) or *N (%)* for every variable

	ITU			PREDO		
	Cord blood	CVS	Placenta (fetal)	Cord blood (EPIC)	Cord blood (450 K)	Placenta (decidual)
Sample size	426	264	486	149	795	139
Gestational age (weeks)	40.04 (1.55)	12.79 (0.82)	39.99 (1.60)	39.87 (1.42)	39.74 (1.67)	39.89 (1.43)
Maternal alcohol use, yes^c^	40 (10)	24 (14)	48 (10)	16 (12)	115 (17)	17 (14)
Maternal smoking, yes^a,b^	18 (4)	29 (11)	20 (4)	13 (9)	32 (4)	13 (9)
Maternal mental disorders, yes	46 (11)	26 (9)	55 (11)	20 (14)	63 (8)	18 (13)
Maternal diabetes, yes^a, c^	93 (22)	57 (22)	105 (22)	26 (17)	222 (28)	20 (14)
Maternal hypertensive disorder, yes^a, b, c^	26 (6)	23 (9)	28 (6)	36 (24)	272 (34)	33 (24)
Maternal BMI^a, b, c^	23.94 (4.21)	24.20 (4.27)	23.82 (4.16)	25.23 (5.76)	27.38 (6.30)	24.85 (5.79)
Maternal age (years)^a, b, c^	34.70 (4.81)	34.96 (5.75)	34.59 (4.86)	32.13 (5.00)	33.33 (5.74)	32.04 (5.17)
Multiparous, yes^b, c^	193 (45)	153 (58)	235 (48)	85 (57)	558 (71)	74 (53)
Induced labor, yes	114 (27)	66 (25)	125 (26)	37 (25)	240 (30)	31 (22)
Delivery mode, aided^a^	129 (30)	87 (33)	145 (30)	51 (35)	233 (30)	55 (40)
Head circumference (cm)	35.10 (1.52)	35.04 (1.73)	35.07 (1.62)	35.21 (1.36)	35.13 (2.15)	35.19 (1.34)
Birth length (cm)^a, b^	50.23 (2.20)	50.13 (2.24)	50.17 (2.40)	49.77 (2.48)	50.21 (2.44)	49.65 (2.53)
Birth weight (g)^a^	3532 (489)	3489 (526)	3534 (509)	3454 (519)	3546 (559)	3425 (523)
Child sex, female	210 (49)	124 (47)	238 (49)	73 (49)	372 (47)	72 (52)

Differences in predictor variables between the ITU and PREDO data sets were tested using t tests for continuous variables and Chi^2^ tests for categorical variables. Variables that showed nominal statistically significant differences (*p* < .05) are indicated as follows:

^a^For difference between ITU placenta vs. PREDO placenta data sets

^b^For difference between ITU cord blood vs. PREDO EPIC cord blood data sets

^c^For difference between ITU cord blood vs. PREDO 450 K cord blood data sets

### Performance of epigenetic clocks in the investigated tissues

We first evaluated the performance of the two epigenetic clocks for cord blood [20, 21] and for placenta [22, 23] in our sample. The clocks differ in the included CpGs, and only share two CpGs (cg07816074, cg16536918; cord blood clocks) with negative weights, and one CpG (cg00307685; placenta clocks) with positive weight, respectively. Nevertheless, we observe high Pearson correlations in DNAm GA between the cord blood clocks (*r* = 0.77, *p* < 0.001 for ITU; *r* = 0.76 *p* < 0.001 for PREDO EPIC data; *r* = 0.51, *p* < 0.001 for PREDO 450 K data), and a medium to high Pearson correlation in the placenta clocks (*r* = 0.44, *p* < 0.001 for ITU; *r* = 0.48, *p* < 0.001 for PREDO). Scatter plots are provided in Additional file 7: Figure S5.

To evaluate the accuracy of an epigenetic clock, three main metrics have been proposed: the average difference between DNAm age and chronological age, the median absolute difference between DNAm age and chronological age, and the correlation between DNAm age and chronological age [4]. As shown in Table 2, the overall accuracy of the clocks was satisfactory, with relatively low median absolute deviations and high Pearson correlations between DNAm age and chronological age (see Additional file 7: Figure S6 for scatter plots). It is evident from these statistics that the estimations were more precise for cord blood as compared to placenta. Furthermore, Bohlin’s clock outperformed Knight’s clock for cord blood and Lee’s clock outperformed Mayne’s clock for placenta for all of the named criteria. Based on this, all following analyses were conducted with Bohlin’s clock for cord blood and Lee’s clock for placenta. Between these clocks, there is no overlap in the underlying CpGs.

**Table 2:** Performance metrics of the four clocks in all available tissues

Cord blood	Bohlin’s clock						Knight’s clock					
	DNAm GA		$\Delta$ DNAm GA			*r*	DNAm GA		$\Delta$ DNAm GA			*r*
	*M*	*SD*	*M*	*SD*	*MAD*		*M*	*SD*	*M*	*SD*	*MAD*	
ITU	39.80	0.93	− 0.23	0.94	0.92	.83*	38.91	1.47	− 1.13	1.19	1.17	.69*
PREDO (EPIC)	39.72	0.84	− 0.16	0.90	0.98	.80*	39.23	1.39	− 0.64	1.05	0.88	.72*
PREDO (450 K)	38.84	1.14	− 0.90	1.19	1.02	.70*	38.44	2.02	− 1.29	1.90	1.55	.48*
	Lee’s clock						Mayne’s clock					
	DNAm GA		$\Delta$ DNAm GA				DNAm GA		$\Delta$ DNAm GA			
Placenta	*M*	*SD*	*M*	*SD*	*MAD*	*r*	*M*	*SD*	*M*	*SD*	*MAD*	*r*
ITU CVS	10.55	1.48	− 2.24	1.14	1.07	.64*	11.69	1.81	− 1.09	1.63	1.57	.43*
ITU Placenta	38.53	1.40	− 1.45	1.41	1.29	.56*	32.68	1.91	− 7.31	1.91	1.73	.28*
PREDO	38.03	1.25	− 1.85	1.24	1.10	.58*	31.69	1.44	− 8.19	1.56	1.63	.41*

*M* = mean; *SD* = standard deviation; *MAD* = median absolute deviation; *r* = Pearson correlation coefficient for DNAm GA and chronological GA; DNAm GA = DNA methylation gestational age; $\Delta$ DNAm GA = raw difference between estimated DNA methylation gestational age and chronological gestational age (measured in weeks)

^*^p < 0.001

### Factors impacting the relative epigenetic age in gestational and perinatal tissues

**Fig. 3:**
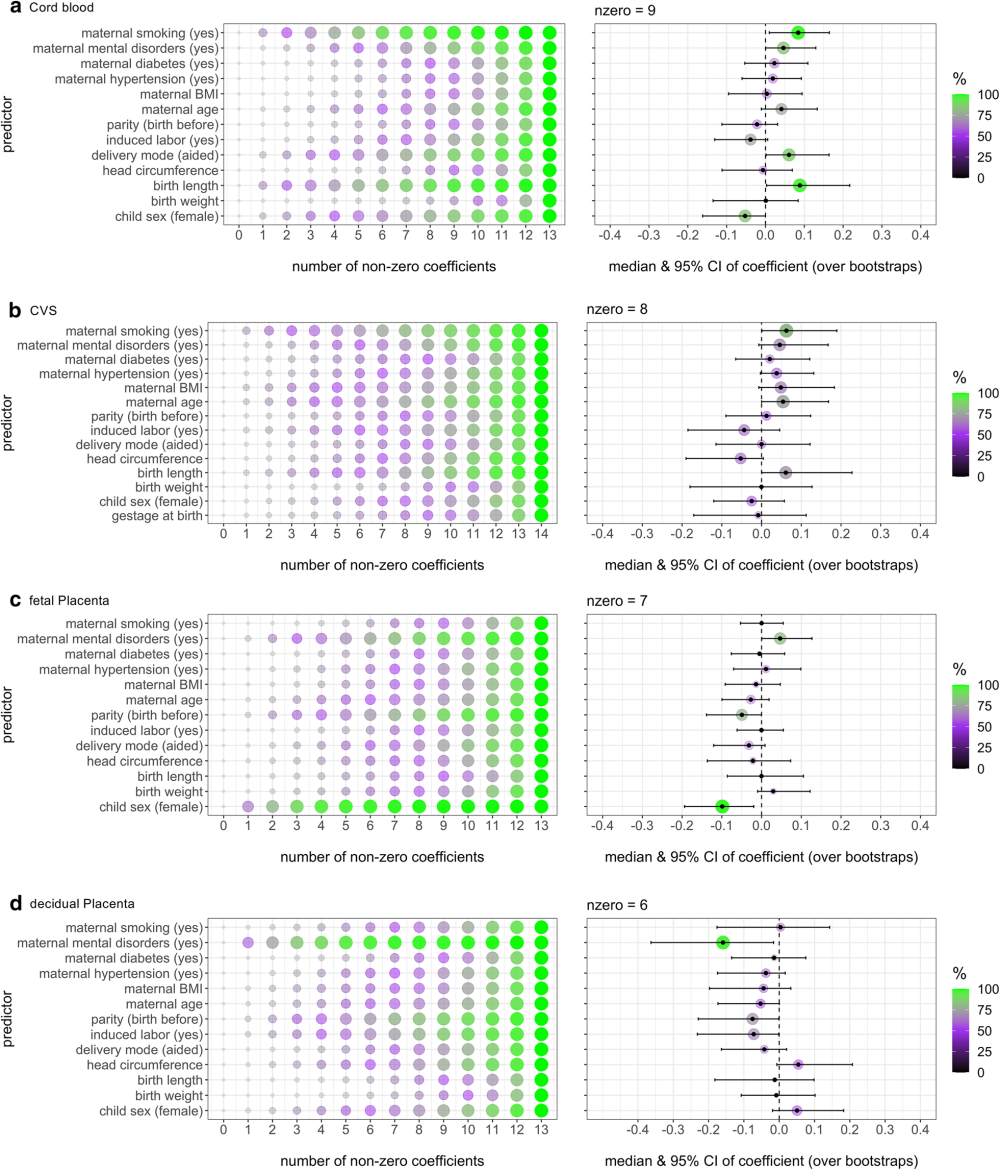
Outcomes of elastic net regression models in different tissues. Associations between birth- and pregnancy-related variables (predictors) and EAAR (adjusted for gestational age at time of sampling, cell types and ancestry-related information). Depicted are the percentages of variable occurrence in bootstrap models with different number of non-zero coefficients (left) and the coefficients of variables in the final model (right) in cord blood from ITU (**a**), CVS from ITU (**b**), fetal placenta from ITU (**c**) and in decidual placenta from PREDO (**d**). The color coding shows the percentage of occurrence of a variable in the model over bootstraps and the size of the circle is proportional

The association between epigenetic age acceleration residuals (EAAR) and birth- and pregnancy-related variables was tested for cord blood, CVS and placenta tissue separately. The results of the elastic net regressions are summarized in Fig. 3, and further statistical parameters can be found in Additional file 9.

#### Analyses in cord blood

##### Cord blood in ITU

Cord blood samples from ITU with full observations were available for 385 newborns. As described previously in the Methods section, nzero (number of non-zero coefficients) of the elastic net model was chosen by finding the most decreasing median cvm (minimum mean cross-validation error) across bootstrap samples. If a predictor was selected in > 75% of bootstrap samples in this model, we declared it as sufficiently stable. For cord blood data from ITU, the model was chosen with nzero = 9, and five variables were selected in a sufficiently stable manner: maternal smoking (97% of bootstrap samples), maternal mental disorders (83%), delivery mode (87%), birth length (95%) and female sex (84%). Maternal smoking, maternal mental disorders, aided delivery and higher birth length were associated with relatively higher EAAR; female sex was associated with relatively lower EAAR (see Fig. 3a).

##### Replication of cord blood findings in PREDO

Cord blood data were available from both cohorts which enabled a test of the performance of these predictors identified in ITU in an independent cohort (PREDO). In PREDO, 144 samples had complete data from the EPIC array, and 766 from the 450 K array. The beta matrix of median coefficients derived from the final model in ITU was used for a prediction of EAAR in PREDO. The one-tailed Pearson correlation between predicted and true EAAR was *r* = 0.24, *p* = 0.002 for the EPIC array and *r* = 0.11, *p* = 0.002 for the 450 K array (Additional file 8: Fig. S7), supporting that the predictors of EAAR identified in the ITU cohort can be predictive for relative epigenetic age acceleration/deceleration in independent cohorts and different array platforms. We then further analyzed the PREDO data sets independently (Additional file 4) and compared the results with those from ITU. The direction of effects between the predictors and EAAR was consistent across cohorts; however, the strength of the associations and most predictive variables varied between data sets.

#### Analyses in placental tissues

##### CVS in ITU

For CVS, 195 samples were available with full information for all predictor variables and EAAR. The elastic net regression model with nzero = 8 was chosen. Maternal smoking was the only variable with non-zero coefficients in more than 75% of the bootstrap models (81%), and associated with relatively higher EAAR (see Fig. 3b).

##### Placenta (fetal) in ITU

For fetal placenta in ITU, 427 complete observations were available, and the model with nzero = 7 was chosen. In this model, three variables had non-zero coefficients in > 75% of the bootstrap models: Child sex (99%), parity (78%) and maternal mental disorders (82%). Maternal mental disorders were associated with relatively higher EAAR, while being multipara and female sex of the child were related to relatively lower EAAR (see Fig. 3c).

##### Placenta (decidual) in PREDO

For decidual placenta, the model could be built from 117 samples, and nzero = 6 was selected. In this model, maternal mental disorders occurred sufficiently stably over the bootstrap samples (96%) and were associated with relatively lower EAAR (see Fig. 3d).

### Complementary analyses

Separate analyses for male and female placentas are described in detail in Additional file 5. These analyses showed that the strength of association of predictors with epigenetic age acceleration/deceleration can differ between males and females. Further, some predictors showed tendencies of different directions of associations between males and females, but as these patterns were not sufficiently stable and strong in our analyses, this needs to be confirmed with larger sample sizes in future studies.

We additionally report analyses including maternal alcohol use (smaller sample sizes, n = 367 in cord blood, n = 133 in CVS, n = 412 in placenta from fetal side (ITU), and n = 106 in placenta from decidual side (PREDO)) in Additional file 6. Overall, maternal alcohol use does not seem to be strongly related to epigenetic age acceleration or deceleration in gestational and perinatal tissues; only a weak association was found with relatively higher EAAR in decidual placenta.

### Cross-tissue analyses

**Fig. 4:**
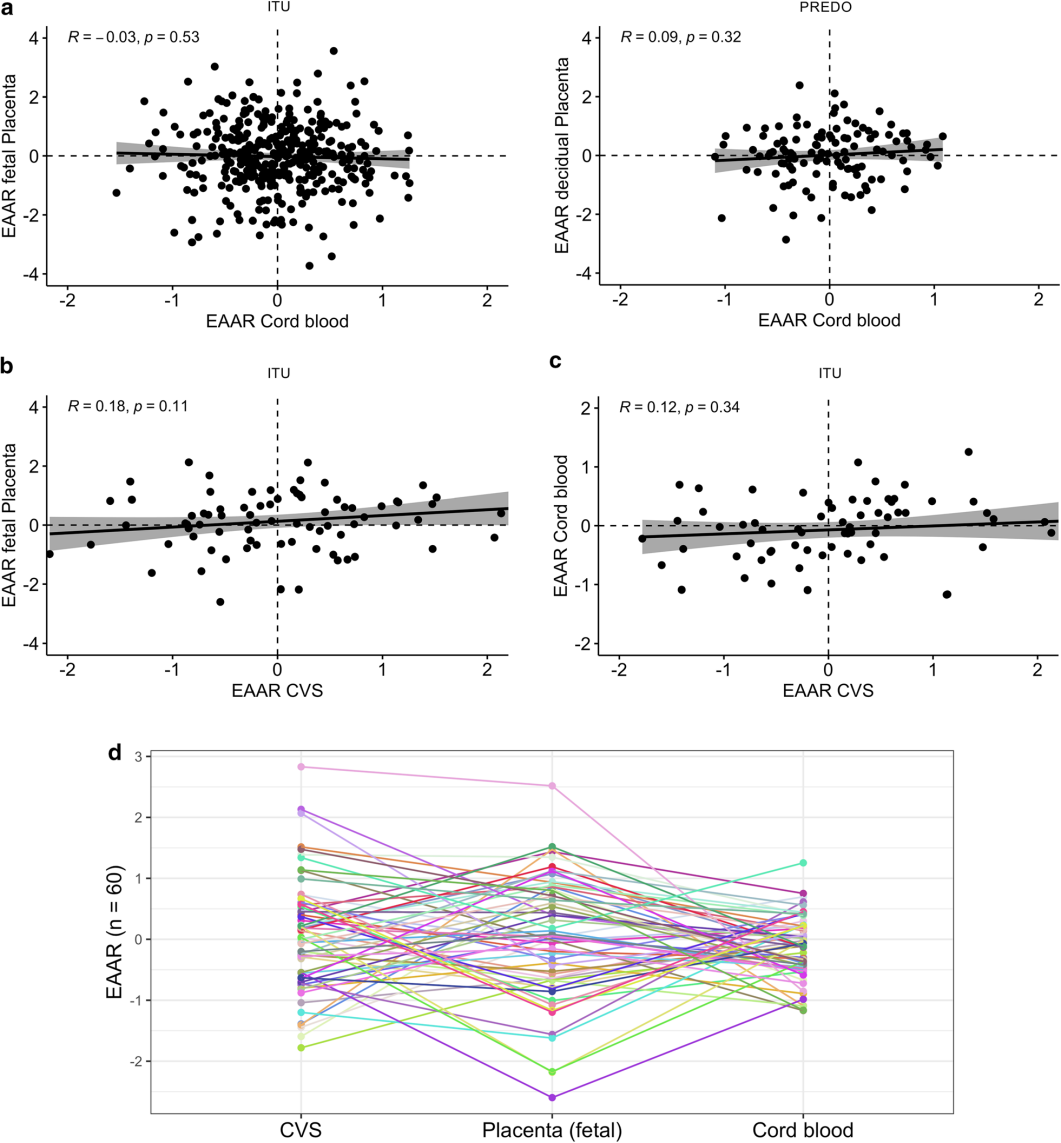
Relationship of epigenetic age acceleration/deceleration between different tissues. In children with more than one tissue available, the relationship of epigenetic age acceleration or deceleration between the respective tissues can be illustrated. Depicted are the scatter plots of EAAR for (**a**) cord blood and placenta from both ITU (n = 363) and PREDO (n = 116), (**b**) CVS and placenta from ITU (n = 78), and (**c**) CVS and cord blood from ITU (n = 66). The regression line is plotted together with a 95% confidence interval, and the Pearson correlation coefficient is depicted. Individual differences in EAARs between CVS, placenta and cord blood from ITU are further illustrated (**d**) for n = 60 children from ITU, where each color represents one individual.

To evaluate how epigenetic age and acceleration or deceleration relate between the tissues, we calculated Pearson correlations between the DNAm GAs and EAARs, respectively. We further tested for statistically significant differences in epigenetic age acceleration/deceleration between tissues using paired Student’s t test or paired Wilcoxon signed-rank test in case of unfulfilled assumptions for the parametric test. We illustrate the differences in EAARs between tissues from the same individuals in Fig. 4. For n = 60 children from ITU with complete tissue data (cord blood, CVS and fetal placenta), we illustrate individual differences in EAAR in Fig. 4d.

#### Cord blood and placenta

The correlation between DNAm GAs of cord blood and placenta was significant in both ITU, *r* = 0.48, *p* < 0.001, and PREDO, *r* = 0.48, *p* < 0.001. This was expected, as the DNAm GA is an estimator of GA, which is the same for these tissues at birth. However, there was no significant correlation between the EAARs, neither in ITU, *r* = − 0.03, *p* = 0.53, nor in PREDO, *r* = 0.09, *p* = 0.32 (Fig. 4a). This suggests that individual epigenetic age acceleration does not correspond between cord blood and fetal placenta, nor between cord blood and decidual placenta. Furthermore, there was no indication of generally higher or lower age acceleration/deceleration in cord blood (*M* = − 0.01, *SD* = 0.49) and fetal placenta (*M* = − 0.02, *SD* = 1.11) from ITU, *t* = 0.16, *p* = 0.88, nor in cord blood (*M* = − 0.01, *SD* = 0.48) and decidual placenta (*M* = 0.01, *SD* = 0.90) from PREDO, *t* = − 0.27, *p* = 0.79.

#### CVS and (fetal) placenta

The correlation between DNAm GAs of CVS (at sampling) and fetal term placenta in ITU was significant *r* = 0.27 *p* = 0.01. However, there was no significant correlation between the EAARs at sampling in CVS and fetal placenta, *r* = 0.18, *p* = 0.11 (see also Fig. 4b). Overall, epigenetic age acceleration/deceleration was not significantly higher or lower in CVS (*M* = 0.03, *SD* = 0.93) versus fetal placenta (*M* = 0.14, *SD* = 1.0), *t* =  − 0.73, *p* = 0.47.

#### CVS and cord blood

Neither the correlation between DNAm GAs of CVS and cord blood in ITU *r* = 0.09, *p* = 0.46, nor the correlation between the EAARs at sampling in CVS and cord blood, *r* = 0.12, *p* = 0.34 was significant (see Fig. 4c). Paired Wilcoxon signed-rank test showed no significant difference in epigenetic age acceleration/deceleration between CVS (*M* = 0.08, *SD* = 0.95) and cord blood (*M* = − 0.07, *SD* = 0.54), *p* = 0.32.

## Discussion

Our analyses uncovered the strength and direction of associations between several birth- and pregnancy-related variables with gestational epigenetic age acceleration or deceleration in CVS, cord blood, fetal and decidual placenta tissue. Further, we showed that the factors related to epigenetic aging differ between the tissues, and that there is no correspondence in individual epigenetic age deviations across these tissues.

### Insights from single tissue analyses

We will first discuss variables that showed associations with epigenetic age deviations. Among the considered child characteristics, we found newborn anthropometric data, especially birth length, to be associated with relatively higher epigenetic age acceleration in cord blood. This is in accordance with two other studies applying Bohlin’s clock [28, 29]. In contrast, anthropometric characteristics of the child seem to be less associated with epigenetic aging in placental tissues. Female child sex was related to relatively lower epigenetic age acceleration in both cord blood and fetal placenta.

Regarding maternal characteristics, smoking during pregnancy was associated with relatively higher epigenetic age acceleration. We observed this in cord blood as well as CVS tissue, but neither in fetal, nor decidual term placenta.

Furthermore, maternal mental disorders showed an association with epigenetic age acceleration in cord blood and in fetal placenta within the ITU cohort. However, in PREDO, maternal mental health disorders were not associated with cord blood epigenetic age, but these disorders were associated with epigenetic age deceleration in decidual placenta. Medical treatment can be of relevance when considering mental diagnoses, for example, the influence of considering SSRIs was reported in a previous study [52]. However, the differences between ITU and PREDO are unlikely to be due to differences in the prevalence or treatment of mental disorders between the samples: the rate of mental disorders was similar across samples and both cohorts had similar access to care through the Finnish healthcare system. In both cohorts, lifetime occurrence of any mental disorder up to childbirth was identified in the same way based on national register data. Nevertheless, some differences between the cohorts remain: while PREDO was enriched for participants with risk factors of pre-eclampsia and intrauterine growth restriction, ITU was enriched for participants who underwent prenatal fetal chromosomal testing. It is possible that these differences in the populations explain some discrepancies between the findings. Furthermore, differences in epigenetic aging may also arise from distinct biological characteristics of the two placental regions with different functions and tissue composition. In fact, cell count estimates between CVS and placenta but also between fetal and decidual placenta showed substantial differences (see Additional file 2). Altogether, our results support the hypothesis that maternal mental disorders associate with epigenetic age deviations in perinatal tissues. We encourage future studies, e.g. with both decidual- and fetal-side samples from the same individuals, to further evaluate tissue specificity.

Another predictor related to the mother and pregnancy was parity, which showed an association with epigenetic age deceleration in fetal placenta. Out of the variables related to the delivery process itself, aided delivery was associated with relatively higher epigenetic age acceleration in cord blood.

Overall, relevant predictors for relative epigenetic age acceleration in gestational and perinatal tissues span the whole spectrum from child and mother to birth and pregnancy characteristics.

Our results indicate that relatively lower or higher epigenetic age deviation cannot be clearly assigned to birth- and pregnancy-related variables that are considered as being more favorable versus unfavorable in the context of disease risk. This suggests that gestational epigenetic age acceleration or deceleration itself may not be linked to a higher risk for diseases per se, but that these associations are more complex and dependent on the condition and tissue during the earliest phase of life. It has been proposed that adjustments to the maturational tempo may explain why children in both favorable and unfavorable environments can exhibit epigenetic age acceleration, as this possibly constitutes specific adaptations to future challenges [53, 54]. Recent studies in adult populations also reported large differences in associations with lifestyle risk factors among studies and clocks [14, 55, 56], and it was assumed that different epigenetic clocks may capture the consequences of different environmental stimuli [14]. Overall, it has to be noted that the mechanistic underpinnings of biological age and epigenetic clocks are still discussed and not fully understood [3, 19, 54].

### Cross-tissue relationships

In addition to looking at factors associated with gestational epigenetic aging in single tissues, we investigated the epigenetic age relationship between tissues. The estimated epigenetic age was congruent between cord blood and placenta, which has also been reported for most tissues investigated in adults so far with only few exceptions [7].

There was no evidence for one tissue being in general epigenetically older or showing remarkable biases toward epigenetic age acceleration or deceleration. However, the relative epigenetic age acceleration or deceleration in the different tissues was not concordant, i.e. a child with relatively high EAAR in one tissue did not necessarily display relatively high EAAR in another tissue (see Fig. 4d). This is in accordance with the fact that we observed different predictors as being the most related to epigenetic aging in the different tissues, and in line with the proposition of different characteristics of epigenetic age acceleration between diverse tissues [19, 57]. Although we can only speculate about the underlying processes at this point, these results suggest that the factors with strongest influence on gestational epigenetic age acceleration and deceleration vary between functionally different parts of one tissue (fetal vs. decidual placenta), developmental stage of the placenta (CVS vs. term placenta), and between placental and cord blood tissues. This indicates that with the currently available epigenetic clocks for specific gestational/perinatal tissues, the epigenetic age of the newborn should be seen as a characteristic linked to the respective tissue, and less as a general characteristic of the child itself. Thus, future health and developmental trajectories associated with gestational epigenetic age can be expected to show a more tissue dependent pattern, too, which should be kept in mind when interpreting results from one tissue. It would be interesting to see if a cross-tissue or phenotypic clock for the gestational and perinatal period, as developed for adults [4, 58], also shows more congruent associations of epigenetic age acceleration and deceleration in newborns with different predictors and outcomes. However, it may also be that tissue-specific effects are generally more pronounced in gestational and perinatal tissues, probably because of the particularly dynamic (epigenetic) processes taking place in these tissues, and therefore especially important to consider and disentangle.

### Strengths and limitations

A major strength of the present study is the inclusion of three different perinatal tissues. Insights into epigenetic age acceleration in CVS are unique, as well as the examination of epigenetic aging across gestational and perinatal tissues. In addition, we were able to compare and contrast tissues from two independent Finnish cohorts. While the context of recruitment for the two studies was different, as elaborated above, the individual predictors were comparable across studies and showed very similar correlation structure (see Table 1 and Fig. 2). To thoroughly assess the impact of the different factors and account for confounding, we chose a modeling approach that enables the inclusion of all variables in one model, can deal with correlations among predictors and performs variable selection [59]. We restricted the analysis to the set of variables which were available for both cohorts and all tissues. On the one hand, this is a strength, as this approach allowed us to identify important predictors of epigenetic aging (in cord blood) in one cohort, and then test these predictors in a second, independent, cohort, to validate the findings. These predictors are also likely to be available in many clinical settings and study cohorts. On the other hand, this approach has its limitations, as there are likely additional factors influencing gestational epigenetic age acceleration/deceleration, which were beyond the scope of the current study. Additional assessments of biological maternal variables, such as hormone levels, immune status and placental functional, could be important to better characterize influences on gestational epigenetic aging. Further, the presented results are of correlative nature, and we refer to perinatal factors as predictors even when they occurred after the measurement of the outcomes, which was done for consistency, modeling reasons and ease of interpretation, but does not imply a causal assumption. Studies in animal models or in vitro may help to better understand in which cases epigenetic age acceleration or deceleration is a cause versus consequence of other factors. Additionally, we did not include any postnatal measures in this analysis. Thus, future studies should test whether epigenetic age deviations in any of these tissues associate with altered health trajectories. Furthermore, investigating the relationship between genetic architecture and epigenetic aging during the gestational period was beyond the scope of the current analysis, but further studies incorporating similar approaches as already used for adults [60, 61] may also provide additional insights for the earliest developmental phase. Apart from this, both cohorts are of Finnish origin, which could reduce the generalizability of findings to other ethnicities and countries with, for example, lower socioeconomic status and prenatal health care, as well as for clinical samples. Despite the relatively large data resource, missing values led to a reduction of sample sizes, and biospecimens for more than one tissue were only available for a smaller proportion of individuals. When considering differences between fetal and decidual placenta, it is necessary to take into account that these samples were not only taken from different sides of the placenta, but also from different individuals and cohorts. Future studies sampling the same placenta from different sides are needed to better understand potential biological differences.

## Conclusions

Our results suggest that factors affecting the deviation between gestational epigenetic and chronological age differ between gestational and perinatal tissues. In addition, more or less favorable birth- and pregnancy-characteristics were not associated with either accelerated or decelerated epigenetic age in a consistent direction. This indicates that both epigenetic age acceleration and deceleration are associated with distinct risk and protective factors, and possibly distinct, tissue specific, developmental trajectories in newborns. In line with this, there is no concordance between epigenetic age acceleration/deceleration in different gestational and perinatal tissues from the same individual. Overall, when using the currently available tissue specific clocks, the epigenetic age of the newborn should be evaluated on the tissue-level rather than on the individual level. Considering this can lead to important insights in health trajectories which may be distinct depending on the epigenetic aging profile of the underlying tissue.

## Supplementary Information

**Additional file 1. Table S1**: CpGs from the epigenetic clocks that were not included due to their missingness on the EPIC array.

**Additional file 2. Table S2**: Mean estimated cell-type proportions (%) and SD in every data set.

**Additional file 3. Figure S1**: Illustration of analysis steps using cord blood data from ITU.

**Additional file 4.** Supplementary analysis. Factors impacting the relative epigenetic age in cord blood from the PREDO cohort.

**Additional file 5.** Supplementary analysis. Factors impacting the relative epigenetic age in placenta, stratified by sex.

**Additional file 6.** Supplementary analysis. Factors impacting the relative epigenetic age, analyses including maternal alcohol use as predictor.

**Additional file 7. Figures S5 and S6.** Fig. S5: Scatter plots illustrating the Pearson correlation between DNAm GA estimated with the available cord blood (a) and placenta (b) clocks. The regression lines are plotted together with a 95% confidence interval and the Pearson correlation coefficients are depicted. Fig. S6: Scatter plots illustrating the Pearson correlation between estimated (DNAm) and chronological GA for Bohlin’s clock (a), Knight’s clock (b), Lee’s clock (c) and Mayne’s clock (d). The regression lines are plotted together with a 95% confidence interval and the Pearson correlation coefficients are depicted.

**Additional file 8. Figure S7**: Scatter plots showing the one-tailed Pearson correlation between EAAR estimated in the PREDO cord blood data sets using the beta matrix of median coefficients derived from the final model in ITU and true EAAR values observed in the PREDO cord blood data sets. The regression lines are plotted together with a 95% confidence interval and the Pearson correlation coefficients are depicted.

**Additional file 9. Table S3**: Median (MED) coefficients, 95% confidence intervals (lcl = lower confidence limit; ucl = upper confidence limit) and % of non-zero of predictor variables across 1000 bootstrap samples for the final elastic net models.

## Data Availability

Due to the sensitive nature of the patient data used in the current study and consent, the data sets are not and cannot be made publicly available. However, an interested researcher can obtain a de-identified data set after approval from the PREDO or ITU Study Board. Data requests may be subject to further review by the national register authority and by the ethical committees.
