# Supplementary material for: Characteristics of epigenetic aging across gestational and perinatal tissues
Source: Clin Epigenetics. 2021 Apr 29;13:97. doi: 10.1186/s13148-021-01080-y (PMC8082803; doi:10.1186/s13148-021-01080-y)

Step 1:

Elastic net regressions were performed with 10-fold cross validation for hyperparameter selection on 1000 bootstrap samples, models with minimum mean cross-validation error (cvm) were chosen for every number of non-zero coefficients (nzero) per bootstrap sample.

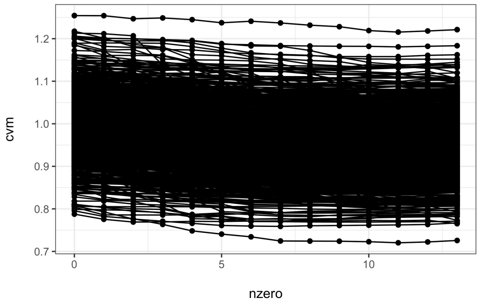

Depending on nzero, variables occur with different frequency over bootstraps.

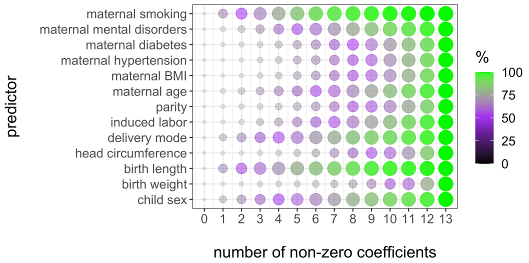

Step 2:

The median cvm over bootstraps for every nzero was plotted. The decision for a final number of nzero can be aided by a function drawing a straight line from the first to the last point of the curve and finding the data point farthest away from this line, i.e. the elbow of the curve with most decreasing cvm. The final number of nzero was chosen and the respective model with its parameters was used for following analysis steps.

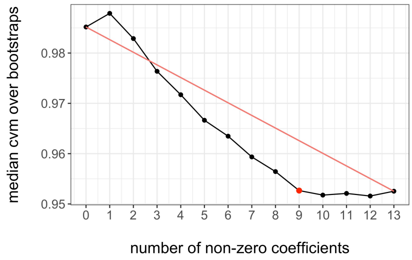

Step 3:

Variable-selection based on a cut-off criterion of occurrence in > 75% of bootstraps.

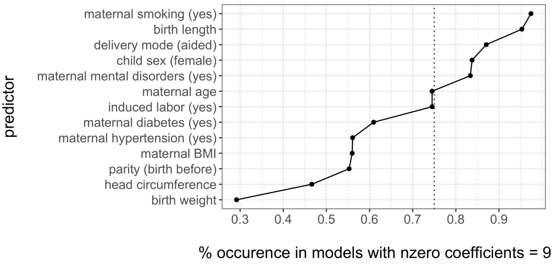

Step 4:

Median coefficients over bootstraps when the predictor was not zero, as well as the 95% confidence interval, were calculated and plotted together with the percentage of occurrence of the predictor over bootstraps. For cord blood, the median coefficients of the selected variables were used for prediction in an independent cohort.

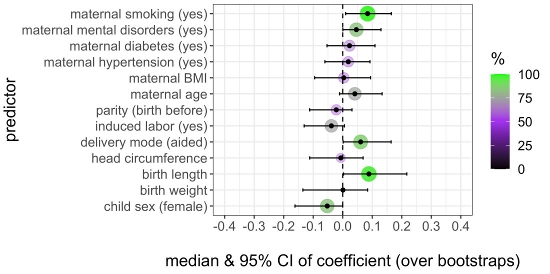

Supplement: Supplementary file 3 — Additional file 3. Figure S1: Illustration of analysis steps using cord blood data from ITU. [file 13148_2021_1080_MOESM3_ESM.pdf]
