# Supplementary material for: Characteristics of epigenetic aging across gestational and perinatal tissues
Source: Clin Epigenetics. 2021 Apr 29;13:97. doi: 10.1186/s13148-021-01080-y (PMC8082803; doi:10.1186/s13148-021-01080-y)
Supplement: Supplementary file 4 — Additional file 4. Supplementary analysis. Factors impacting the relative epigenetic age in cord blood from the PREDO cohort. [file 13148_2021_1080_MOESM4_ESM.docx]

**Additional file 4.**

**Factors impacting the relative epigenetic age in cord blood from the PREDO cohort.**

To further check if the direction of effects found in ITU can also be seen in an independent analysis in cord blood from PREDO, we performed the same elastic net analysis in these data sets.

In samples with complete information from the EPIC array (n = 144), the model was selected with nzero = 7. Four variables occurred in > 75% of bootstraps: maternal smoking (78%), induced labor (82%), parity (80%) and head circumference (83%). As in ITU, maternal smoking was associated with relatively higher EAAR (Fig. S2a). Additionally, head circumference was associated with relatively higher EAAR. Although birth length and aided delivery were not as predictive in PREDO as in ITU, the direction of effects are in the same direction (associated with higher EAAR). Parity and induced labor were both associated with relatively lower EAAR.

In samples from the 450K array (n = 766) the model was selected with nzero = 6. Birth length (89%) and parity (86%) occurred sufficiently stable (Fig. S2b). As in ITU cord blood data, higher birth length was associated with relatively higher EAAR. Given birth before was, congruent with the PREDO EPIC data set, associated with relatively lower EAAR.

**Fig. S2. Associations between birth- and pregnancy-related variables and epigenetic age acceleration/deceleration in PREDO cord blood data.**

**
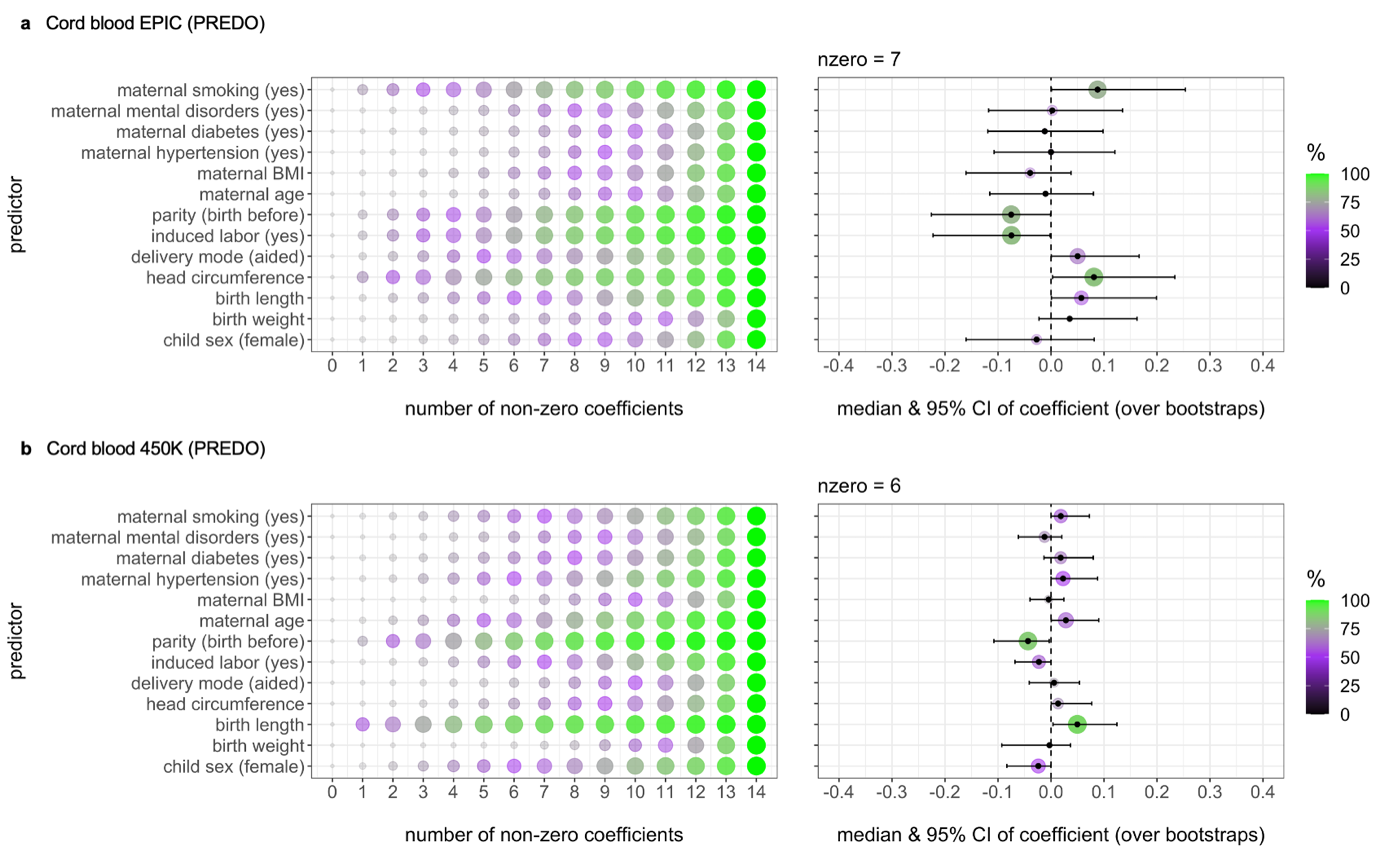
**

Associations between birth- and pregnancy-related variables (predictors) and EAAR (adjusted for gestational age at time of sampling, cell types and ancestry-related information). Depicted are the percentages of variable occurrence in bootstrap models with different number of non-zero coefficients and the coefficients of variables in the final model in cord blood from PREDO derived from EPIC array (**a**) and 450K array (**b**).
