# Supplementary material for: Characteristics of epigenetic aging across gestational and perinatal tissues
Source: Clin Epigenetics. 2021 Apr 29;13:97. doi: 10.1186/s13148-021-01080-y (PMC8082803; doi:10.1186/s13148-021-01080-y)
Supplement: Supplementary file 5 — Additional file 5. Supplementary analysis. Factors impacting the relative epigenetic age in placenta, stratified by sex. [file 13148_2021_1080_MOESM5_ESM.docx]

**Additional file 5.**

**Factors impacting the relative epigenetic age in placenta, stratified by sex.**

Placentas from male and female newborns were considered in separate models to evaluate if any interactions between sex and the other predictors might be present.

We had full information from 218 male and 209 female fetal placenta samples from ITU (sampled from the fetal side of the placenta). In the male’s placenta model, nzero = 5 was selected. No variable occurred sufficiently stable (>75%) over bootstraps in this model. In the female’s placenta model, the model with nzero = 7 was selected. Here, two predictors were not zero in > 75% of the bootstrap models: delivery mode (97%) and parity (94%). Aided delivery, as well as a previous birth were associated with relatively lower EAAR (Fig. S3a). Comparing these results to those of the combined model in fetal placenta (Fig. 3c), it can be seen that the effects in the combined model seem to be driven more strongly by the effects found in females.

Regarding placenta sampled from the decidual side (PREDO), full observations were available for 56 males and 61 females. The final model for placentas from male children was chosen with nzero = 5. Only maternal mental disorders until child birth were sufficiently predictive over bootstraps (93%), and associated with relatively lower EAAR (Fig. S3c). For female placentas, nzero = 6 was selected. Here, aided delivery was associated with relatively lower EAAR (Fig. S3d) in a majority of bootstrap samples (91%). The association with maternal mental disorders was already found in the combined model (Fig. 3d), and the direction of effect was similar in males and females. However, aided delivery appears to be only predictive of EAAR in females and even shows an effect of opposite direction in males.

In summary, these analyses show that regarding some variables, associations with placental epigenetic age acceleration/deceleration can vary between the sexes. However, it should be noted that the sample sizes for these models were relatively low, and although the modeling approach prevents from overfitting and strong influences of the underlying data, it may be that due to the smaller sample size and some unbalanced predictors this analysis is less generalizable.

**Fig. S3. Associations between birth- and pregnancy-related variables and epigenetic age acceleration/deceleration, stratified by sex.**

**
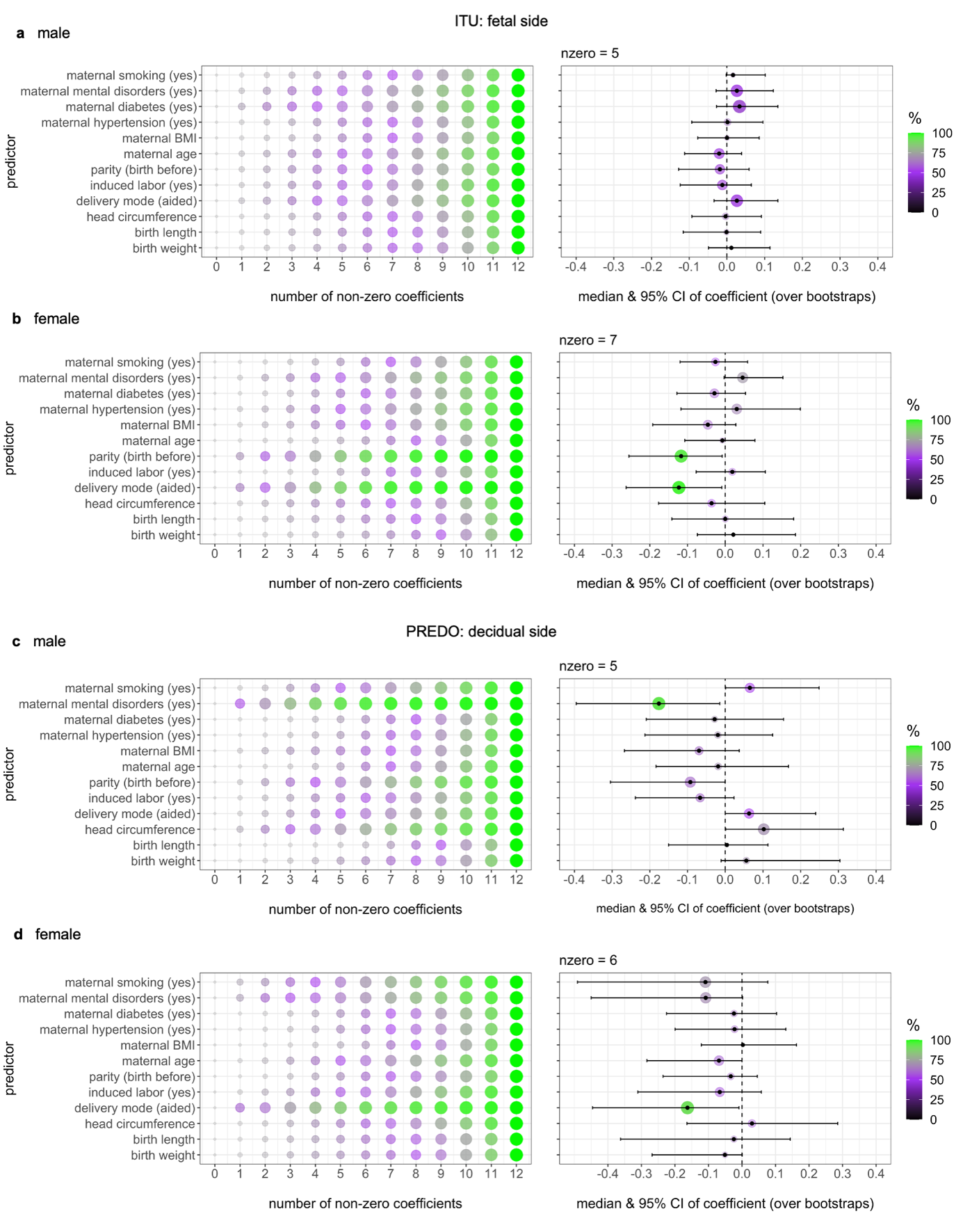
**

Associations between birth- and pregnancy-related variables (predictors) and EAAR (adjusted for gestational age at time of sampling, cell types and ancestry-related information) stratified by sex. Shown are the percentages of variable occurrence in bootstrap models with different number of non-zero coefficients and the coefficients of variables in the final model for fetal placenta from ITU (**a, b**) and decidual placenta from PREDO (**c, d**).
