# Supplementary material for: Characteristics of epigenetic aging across gestational and perinatal tissues
Source: Clin Epigenetics. 2021 Apr 29;13:97. doi: 10.1186/s13148-021-01080-y (PMC8082803; doi:10.1186/s13148-021-01080-y)
Supplement: Supplementary file 6 — Additional file 6. Supplementary analysis. Factors impacting the relative epigenetic age, analyses including maternal alcohol use as predictor. [file 13148_2021_1080_MOESM6_ESM.docx]

**Additional File 6.**

**Factors impacting the relative epigenetic age, analyses including maternal alcohol use as predictor.**

We provide information on additional models for every tissue including maternal alcohol use, as it was assessed in both cohorts but only available for some mothers, leading to relatively large reductions in sample sizes of the models (n = 62 samples for CVS, n = 28 samples for cord blood, n = 15 samples for fetal placenta, n = 11 samples for decidual placenta).

Cord blood samples from 367 newborns from ITU had complete information, of these 10.4% of mothers indicated the use of alcohol while they were pregnant. In the model including maternal alcohol use, nzero = 7 was chosen. Three variables were sufficiently stable over bootstraps: birth length (81%), delivery mode (80%) and maternal smoking (97%). All of these were associated with relatively higher EAAR (Fig. S4a). The effect directions are concordant with the analysis not including maternal alcohol use.

For CVS, 133 samples could be included, and 14.3% of these mothers consumed alcohol at some time during pregnancy. The model with nzero = 10 was selected. Five variables occurred frequently in bootstrap models (Fig. S4b): birth length (94%), head circumference (98%), delivery mode (88%), child sex (81%) and maternal smoking (88%). Maternal smoking and birth length were associated with relatively higher EAAR. Aided delivery, female sex and head circumference were associated with relatively lower EAAR. Maternal smoking occurred sufficiently stable in the model without maternal alcohol use, too (see Fig. 3b). For the other variables, the direction of effect was generally similar in the model without maternal alcohol use, but they did not occur stable enough in the main model (see Fig. 3b).

The model from fetal placenta samples (ITU) was built from 412 samples, with 10.4% of mothers who reported alcohol use, and the final model was chosen with nzero = 6. Most predictive was child sex (95%) – female sex was associated with relatively lower EAAR (Fig. S4c), as it was already seen in the main model (see Fig. 3c).

For placenta from decidual side (PREDO) 106 samples with full information were available, and 12.3% of mothers reported alcohol consumption. The final model was picked with nzero = 9. Five variables had non-zero coefficients over sufficient bootstrap models: child sex (82%), induced labor (81%), parity (87%), maternal mental disorders (92%) and maternal alcohol use (76%). Associated with relatively higher EAAR were maternal alcohol use and female child sex. Maternal mental disorders, given birth before and induces labor were associated with relatively lower EAAR (Fig. S4d). The predictors showed effects in the same direction in the main model, but only maternal mental disorders until child birth occurred sufficiently often over bootstraps there (see Fig. 3d). The effect found for maternal alcohol use should be treated with care in view of the confidence interval including both positive and negative coefficients.

Overall, maternal alcohol use does not seem to be strongly related to epigenetic age acceleration or deceleration in gestational and perinatal tissues, although larger sample sizes and a better balance between mothers consuming alcohol or not would be advantageous to confirm these results.

**Fig. S4. Associations between birth- and pregnancy-related variables (including maternal alcohol use) and epigenetic age acceleration/deceleration.**

**
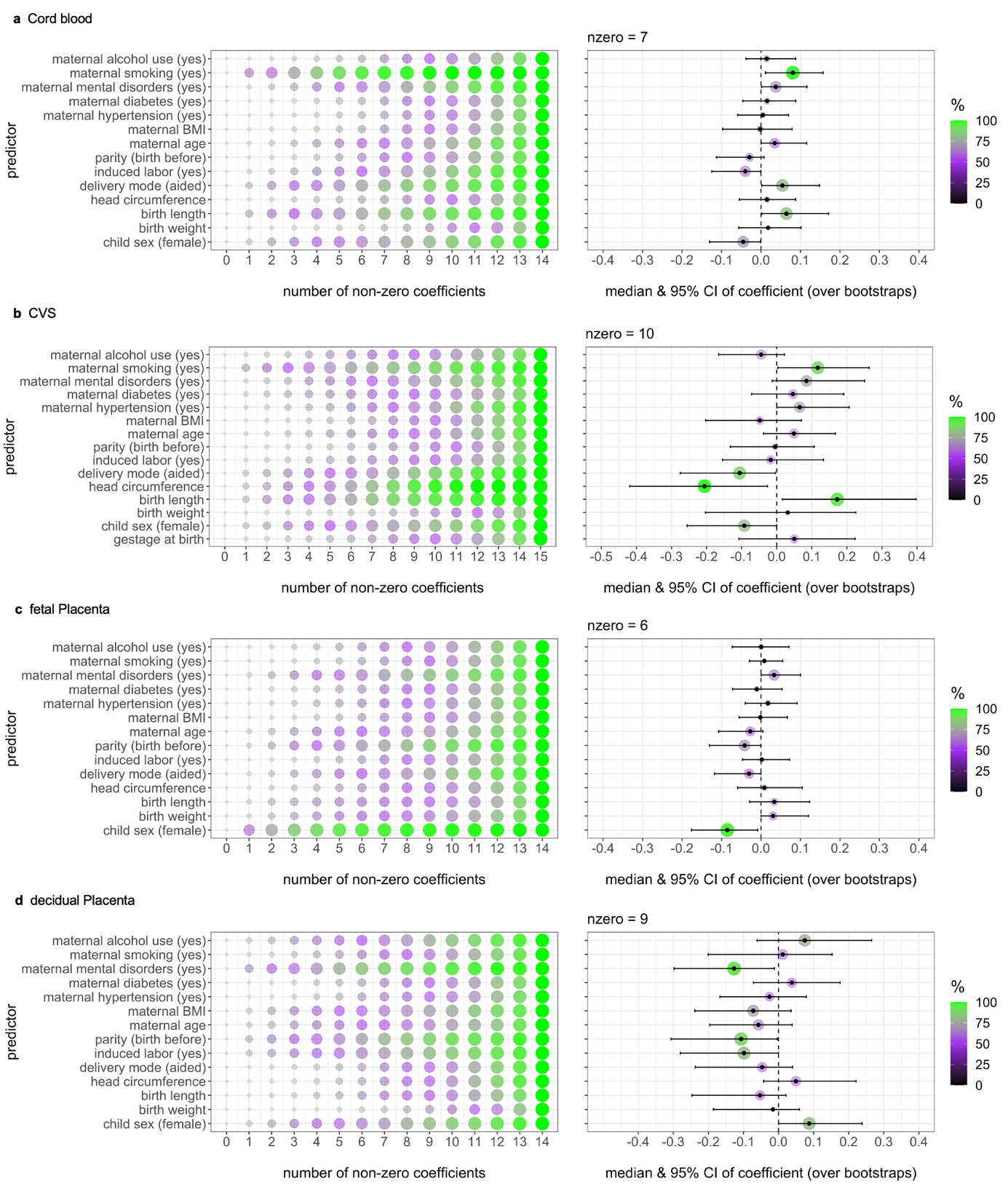
**

Associations between birth- and pregnancy-related variables (predictors), including maternal alcohol use, and EAAR (adjusted for gestational age at time of sampling, cell types and ancestry-related information). Depicted are the percentages of variable occurrence in bootstrap models with different number of non-zero coefficients and the coefficients of variables in the final models for cord blood from ITU (**a**), CVS from ITU (**b**), fetal placenta from ITU (**c**) and decidual placenta from PREDO (**d**).
