# Supplementary material for: Characteristics of epigenetic aging across gestational and perinatal tissues
Source: Clin Epigenetics. 2021 Apr 29;13:97. doi: 10.1186/s13148-021-01080-y (PMC8082803; doi:10.1186/s13148-021-01080-y)

Fig. S5.

a Cord blood clocks

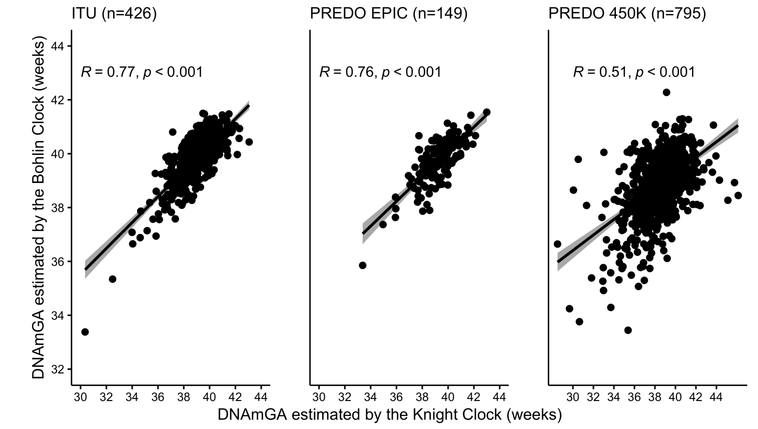

b Placenta clocks

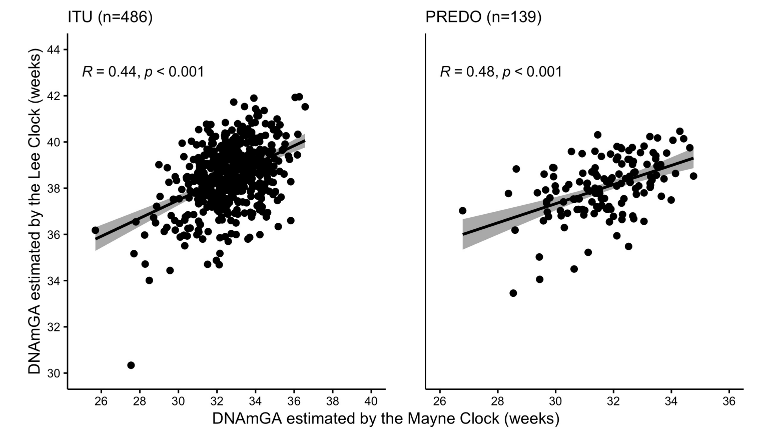

Fig. S6.

a Bohlin's clock

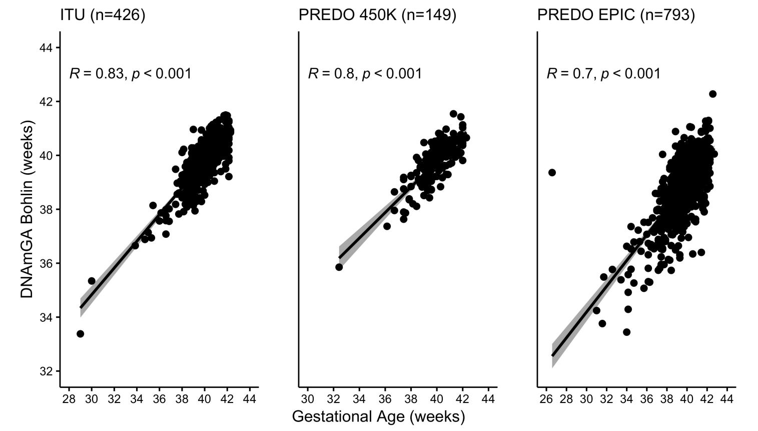

b Knight's clock

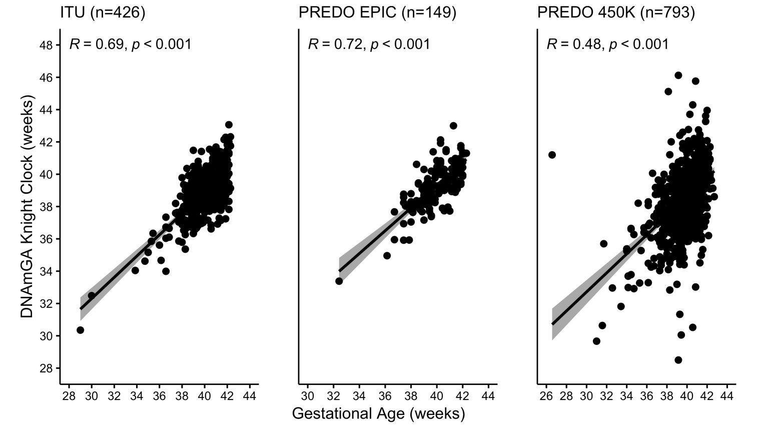

c Lee's clock

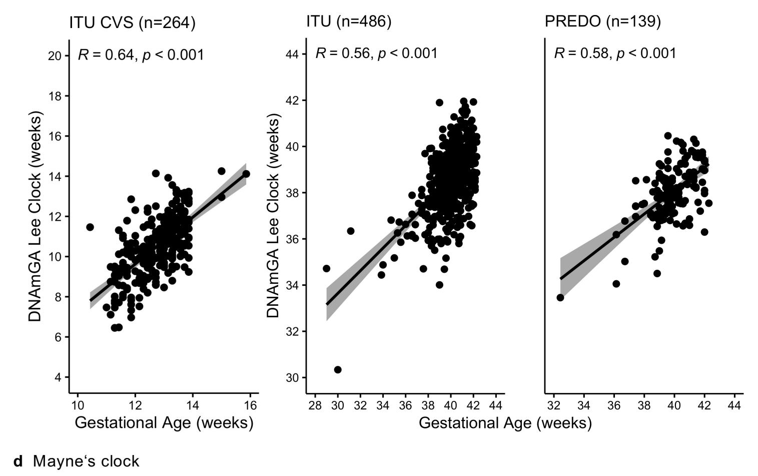

d Mayne's clock

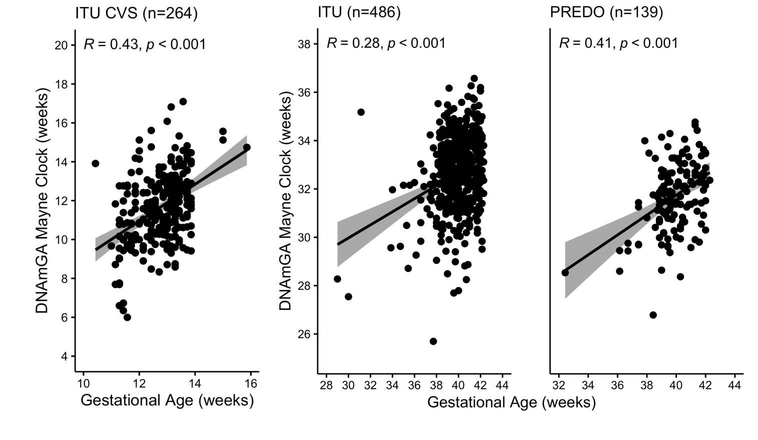

Supplement: Supplementary file 7 — Additional file 7. Figures S5 and S6. Fig. S5: Scatter plots illustrating the Pearson correlation between DNAm GA estimated with the available cord blood (a) and placenta (b) clocks. The regression lines are plotted together with a 95% confidence interval and the Pearson correlation coefficients are depicted. Fig. S6: Scatter plots illustrating the Pearson correlation between estimated (DNAm) and chronological GA for Bohlin’s clock (a), Knight’s clock (b), Lee’s clock (c) and Mayne’s clock (d). The regression lines are plotted together with a 95% confidence interval and the Pearson correlation coefficients are depicted. [file 13148_2021_1080_MOESM7_ESM.pdf]
