# Supplementary figures and images for: Characteristics of epigenetic aging across gestational and perinatal tissues
Source: Clin Epigenetics. 2021 Apr 29;13:97. doi: 10.1186/s13148-021-01080-y (PMC8082803; doi:10.1186/s13148-021-01080-y)

**Fig. S7**

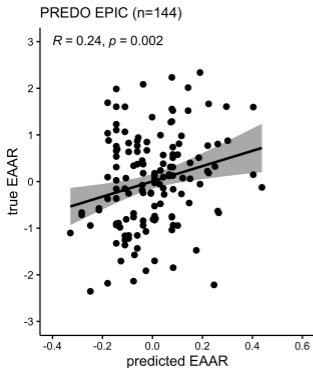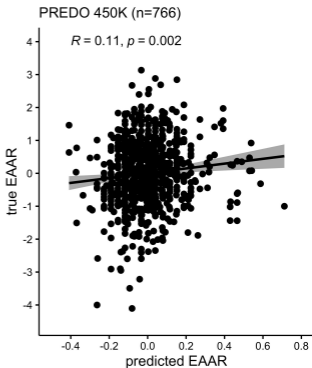

Supplement: Supplementary file 8 — Additional file 8. Figure S7: Scatter plots showing the one-tailed Pearson correlation between EAAR estimated in the PREDO cord blood data sets using the beta matrix of median coefficients derived from the final model in ITU and true EAAR values observed in the PREDO cord blood data sets. The regression lines are plotted together with a 95% confidence interval and the Pearson correlation coefficients are depicted. [file 13148_2021_1080_MOESM8_ESM.pdf]
